# Influence of homozygosity on genomic structural variation analyses for predicting ACL rupture risk in the Labrador Retriever and Rottweiler

**DOI:** 10.3389/fgene.2026.1875383

**Published:** 2026-09-03

**Authors:** Mehdi Momen, Faris Hazim Mohamed Zaimir, Brian W. Davis, Susannah J. Sample, Peter Muir

**Affiliations:** 1 Department of Surgical Sciences, School of Veterinary Medicine, University of Wisconsin-Madison, Madison, WI, United States; 2 Department of Computer Sciences, School of Computer, Data & Information Sciences, University of Wisconsin-Madison, Madison, WI, United States; 3 Department of Veterinary Integrative Biosciences, College of Veterinary Medicine and Biomedical Sciences, Texas A&M University, College Station, TX, United States

**Keywords:** anterior cruciate ligament rupture, breed-specific genetics, dogs, genetic risk prediction, genomic structural variation, runs of homozygosity

## Abstract

**Introduction:**

Anterior cruciate ligament (ACL) rupture is a common orthopaedic disease in dogs, with varying prevalence and genetic susceptibility across different breeds. Here we investigate the association between genomic structural variation (SV) and ACL rupture risk in the Labrador Retriever and Rottweiler breeds.

**Methods:**

We used 1,058 Labrador Retrievers (464 cases, 594 controls) and 108 Rottweilers (83 cases, 25 controls). Using breed‐specific de novo genome assemblies, we first characterized SV between Labrador Retriever and Rottweiler genomes to provide genomic context for breed-level differences. We then quantified runs of homozygosity (ROH) and evaluated the association between homozygosity measures (nROH, AVGROH, and F_ROH_) and ACL rupture risk using breed‐specific and combined‐breed logistic regression models.

**Results:**

The association between homozygosity parameters and ACL rupture in the Labrador Retriever was not significant (*P* > 0.05 for all models). In contrast, inbreeding coefficient (F_ROH_) was significantly associated with increased ACL rupture risk (OR = 2.011, 95% CI:1.120‐3.98, *P*‐value = 0.026) in the Rottweiler when sex and interaction effects were included in the model.

**Conclusion:**

These findings suggest a potential association between inbreeding and ACL rupture risk in Rottweilers. In addition, joint analysis of Labrador Retriever and Rottweiler data revealed multicollinearity between breed and homozygosity content, which highlights the heterogeneity of genetic risk factors across breeds. Our findings suggest that breed‐specific genetic models are crucial for understanding the genetic contribution to ACL rupture and for developing accurate genetic risk prediction tools. Given the relatively small and imbalanced Rottweiler dataset, independent validation in larger populations is warranted to further explore the potential breed‐specific genetic loci contributing to ACL rupture in the Rottweiler.

## Introduction

The development history of domestic dog breeds along with current breeding strategies is increasingly recognized as a contributing factor to genetic diversity, compromised health, and animal welfare (Cruz et al., 2008; Letko et al., 2023). From an evolutionary perspective, the genomes of modern purebred dogs are a patchwork of different regions with some showing short selective sweeps linked to domestication and others displaying longer regions due to recent breed-specific selective sweeps, while the remainder of the genome has undergone a relaxation of selection pressures as the influence of natural and sexual selection has diminished in purebred dogs (Björnerfeldt et al., 2006; Parker, 2012; Shearin and Ostrander, 2010). Structural variation (SV) in the canine genome represents large chromosomal events or genetic variation (≥50bp) including insertions and deletions (InDels), inversions, duplications, translocations and more complex rearrangements that are distributed across the genome (Meadows et al., 2023; Parker, 2012). SVs can directly affect gene dosage, indirectly alter gene expression, unmask recessive alleles, lose regulatory elements, and affect evolution of new genes. These genomic mosaics are associated with processes like phenotypic evolution, disease susceptibility and environmental adaptations (Wang et al., 2019).

In recent years, single nucleotide polymorphism (SNP)-based genotyping arrays and whole-genome sequencing (WGS) data have been used to examine within- and across-breed genomic SVs (Chen et al., 2016; Zhang et al., 2009). Most of these studies analyze runs of homozygosity (ROH), copy-number variation (CNV), insertions, inversions, and translocations to estimate the extent of inbreeding, investigate breed-specific SVs, and gain insights into population diversity of a breed, genome evolution and adaptation, development of certain diseases, and morphological variation (Eggertsson et al., 2019; Hujoel et al., 2022; Meadows et al., 2023).

The genetic basis of canine ACL rupture has been extensively investigated over the past decade through genome-wide association studies, copy number variation analyses, heritability estimation, genomic prediction, and more recently cross-species comparative analyses with humans (Baker et al., 2017; Baker et al., 2020; Baker et al., 2021; Binversie et al., 2020; Momen et al., 2025). However, the contribution of genome-wide homozygosity and inbreeding to disease susceptibility remains poorly understood. SVs play significant roles in genome evolution and are linked to diseases like ACL rupture in specific dog breeds by altering genes related to musculoskeletal structure and function. CNVs associated with key genes involved in ligament strength, joint stability, or inflammation pathways might affect the structural integrity of the ACL and the physiological responses to ligament fiber microinjury (Binversie et al., 2020). Duplications or deletions near regulatory elements of genes involved in collagen production or tissue repair could increase the likelihood of rupture due to imbalances in gene expression (Bell et al., 2012).

Understanding how genetic variation may affect ligament mechanical properties and physiological homeostasis can provide insights into the genetic contribution to risk of ACL rupture, particularly in breeds prone to musculoskeletal disorders, such as the Labrador Retriever or the Rottweiler. Recent studies have shown that predictive polygenic risk score (PRS) models that incorporate both genomic and clinical data could greatly improve identification of dogs with elevated risk of ACL rupture and enable targeted prevention strategies, such as selective breeding and personalized medical care (Baker et al., 2020; Baker et al., 2021; Momen et al., 2025; Miranda et al., 2025). Machine learning and Bayesian regression models, which can leverage complex genomic and phenotypic data, have shown promise in accurately predicting ACL rupture risk in dogs (Baker et al., 2020, Miranda et al., 2025). By developing these advanced statistical models, researchers can work towards a more personalized and precision-based approach to managing ACL rupture in dogs (Baker et al., 2020, Miranda et al., 2025). In follow-up to earlier work (Binversie et al., 2020), we aimed to characterize structural variation between Labrador Retriever and Rottweiler reference genomes and investigate whether genome-wide homozygosity, measured through runs of homozygosity (ROH) and F_ROH_, is associated with ACL rupture risk. We hypothesized that increased homozygosity would be associated with increased disease susceptibility. Structural variation analyses were performed to provide genomic context for breed-specific genomic differences, whereas ROH-derived measures were used in the disease-risk association analyses.

## Methods

### Approach

**FIGURE 1:**
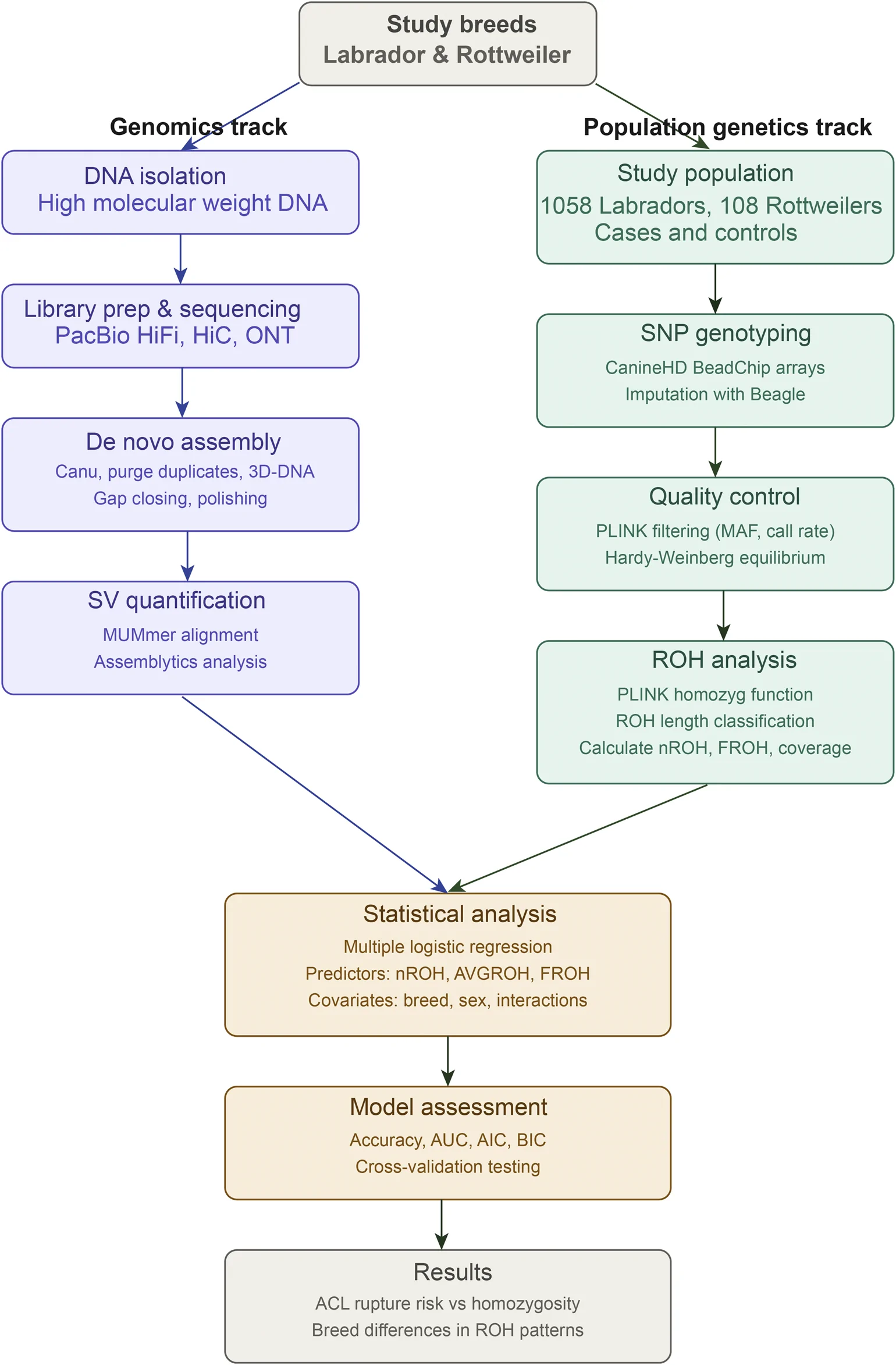
The flowchart of the complete methodology workflow for investigating the relationship between ACL rupture risk and homozygosity in Labrador Retrievers and Rottweilers.

We used the following workflow for the study. Reference genomes were built for the Labrador Retriever and Rottweiler for whole genome alignment and analysis of genomic SV. Groups of Labrador Retriever and Rottweiler were SNP genotyped for ROH analysis. Finally, the relationship between risk of ACL rupture and homozygosity was investigated (Figure 1).

### Labrador Retriever and Rottweiler reference genome assembly

This work was conducted at the University of Wisconsin-Madison Biotechnology Center DNA Sequencing Facility.

High molecular weight DNA isolation. The PacBio Nanobind CBB DNA Kit was used to isolate high molecular weight DNA from whole blood. DNA quality was measured using a NanoDrop instrument (ThermoFisher Scientific, Waltham, MA) by assessing 260/230 and 260/280 ratios. Quantification of the extracted DNA was measured using the Qubit™ dsDNA High Sensitivity kit (ThermoFisher Scientific, Waltham, MA). Samples were diluted before assessing DNA sizing and quality using the Agilent FemtoPulse System (Agilent, Santa Clara, CA).

DNA library preparation and sequencing. A Pacific Biosciences (PacBio) HiFi library was prepared and sequenced according to PN 102-166-600 Version 04 (Pacific Biosciences). Modifications include shearing with Covaris gTUBEs and size selecting with Sage Sciences BluePippin. Library quality was assessed using the Agilent FemtoPulse System. Library was quantified using the Qubit™ dsDNA High Sensitivity kit. The library was sequenced on a Sequel II using Sequel Polymerase Binding Kit 2.2.

For HiC library preparation and sequencing, a freshly collected blood sample was used for crosslinking and HiC library preparation. The Arima-HiC User Guide for Mammalian Blood (Document A160133 v00, Arima Genomics) was followed. Libraries were sequenced on an Illumina NovaSeq 2x150 (Illumina).

For ONT library preparation and sequencing, high molecular weight DNA was isolated from whole blood using Nanobind CBB Big DNA Kit (Circulomics) using the manufacturer’s UHMW DNA Extraction Protocol. DNA purity and concentration were measured on a NanoDrop™ One instrument (ThermoFisher Scientific, Waltham, MA). DNA concentration was verified using the Qubit™ dsDNA HS Assay Kit (ThermoFisher Scientific). DNA size, integrity, and quality were assessed on the Agilent Technologies Femto Pulse System (Agilent, Santa Clara, CA). For some sequencing runs, libraries were prepared using SQK-RAD004 or SQK-LSK109 (Oxford Nanopore Technologies) according to the manufacturer’s recommendations. Libraries were quantified using the Qubit™ dsDNA High Sensitivity kit (ThermoFisher Scientific). Libraries were then sequenced on FLO-MIN106 (R9.4.1) flow cells using a GridION device. For some SQK-RAD004 libraries, DNA was sheared by aspirating and expelling it through a 26-gauge blunt needle 5 times before library preparation.

de novo genome assembly. The canu assembler (ver2.1.1) (Koren et al., 2017) was used to generate a draft assembly using PacBio and ONT reads. Next duplicate haplotigs were purged using purge_dups (https://github.com/dfguan/purge_dups). Hi-C scaffolding of the draft assembly was then performed using 3D-DNA (Dudchenko et al., 2017). Gaps in the scaffolding were then closed with TGS-Gap-closer (Xu et al., 2020) using two rounds of gap closing. For the initial round, the ONT reads were used and for the second round the PacBio continuous long reads (CLR) were used. In a final step, genome polishing was performed using NextPolish (Hu et al., 2020) to correct errors in the base calling using the short paired-end Illumina reads. The quality of the genome assembly was assessed using the Quality Assessment Tool for Genome Assemblies QUAST ver5.3.0 (Mikheenko et al., 2018) and BUSCOv5 (Manni et al., 2021). Labrador Retriever and Rottweiler chromosomal scaffolds were aligned against canFam4.0 (UU_Cfam_GSD_1.0) and renamed and placed in chromosomal order. The Labrador Retriever and Rottweiler reference genomes were then aligned to identify breed related SV.

### Structural variation quantification

To identify and quantify SVs between the genomes of the Labrador Retriever and Rottweiler breeds, we used Assemblytics (https://assemblytics.com/), a web-based tool designed for detecting variation in genome assemblies (Nattestad and Schatz, 2016). The analysis was performed in two steps: (1) Alignment generation began by aligning the Labrador Retriever genome to the Rottweiler reference genome using MUMmer’s *nucmer* program (Kurtz et al., 2004). The alignment was generated with the following parameters: maxmatch, -l 100, and -c 500. This alignment output was a delta file, which served as the input for Assemblytics, and (2) Variant identification: Assemblytics processed the alignment delta file using a plane-sweep algorithm to identify overlapping alignments relative to the sample contig sequence. These alignments were then filtered to retain only the variations with a minimum of 10 kb of unique contig sequence anchor, ensuring high confidence in the variants identified. This filtering step is analogous to the delta-filter component of *dnadiff* (Phillippy et al., 2008) but ensures uniqueness in alignment selection. Finally, the SVs detected by Assemblytics were quantified and categorized by chromosome. The relationship between chromosome length and SV frequency was further explored by plotting the count of each SV type against the sequence length of each chromosome. Correlation analysis was used to assess the strength of the correlation between chromosome length and the number of SVs, with Pearson correlation coefficients (r) reported for each SV type.

### Study population

Genetic information of 1,058 Labradors (464 ACL rupture cases, 594 controls), and 108 Rottweilers (83 ACL rupture cases, 25 controls) were used in our analysis. Control dogs were ≥8 years with no palpable knee laxity on orthopaedic patient exam and knee radiographs with no evidence of effusion or osteophytosis consistent with ACL rupture (Momen et al., 2025).

This dataset consisted of client-owned dogs that were recruited between 2014 and 2025 by the Comparative Orthopaedic and Genetics Laboratory at the University of Wisconsin-Madison. Some Labrador Retrievers were also recruited at Cornell University (Momen et al., 2025). Of the 1,058 Labradors, 52 were part of an independent test set (24 ACL rupture cases, 28 controls) (Miranda et al., 2025). After quality control, 1,006 Labrador Retrievers remained. One additional case dog was excluded because no ROH segments meeting the predefined criteria were detected, resulting in a final dataset of 1,005 Labrador Retrievers. Similarly, six Rottweilers (three cases and three controls) were excluded, resulting in a final dataset of 102 Rottweilers. All procedures were performed in accordance with the recommendations in the Guide for the Care and Use of Laboratory Animals of the National Institutes of Health and the American Veterinary Medical Association and Institutional Animal Care and Use Committee approval (Protocols V1070, V5463 for dogs recruited at the University of Wisconsin-Madison). All owners gave informed consent.

Genotyping was performed using the Illumina CanineHD BeadChip (∼172,000 SNPs, or ∼230,000 canfam3.1 SNPs for more recent samples) or the Thermofisher CanineHD Array (∼710,000 canfam3.1 SNPs). Missing genotypes were imputed using Beagle software (Browning et al., 2021) and quality control was performed using PLINK v1.962 (Chang et al., 2015). SNPs were removed from the dataset if they had minor allele frequency (MAF) <0.01, SNP genotyping call rate <95%, individual dog call rate of <90%, or did not conform to Hardy-Weinberg proportions at a *P* value <1E-06. After quality control, 142,071 SNPs remained for analysis, and 9,903 SNPs were filtered out because they did not fit with Hardy-Weinberg proportions.

### Runs of homozygosity analysis

The number and length of ROH segments can provide insight into both individual and population history. Long ROH segments, typically over 10Mbp, indicate recent inbreeding events occurring within about the last five generations. We assumed that if the Rottweiler has longer or more numerous ROH regions associated with ACL rupture compared with the Labrador Retriever, this might suggest higher genetic predisposition to ACL rupture due to inbreeding.

We used PLINK v1.9 software (Chang et al., 2015) to analyze SNP genotypes for runs of homozygosity (ROH) in both dog breeds using the--homozyg function. The ROH detection process involved a three-step scanning window approach. A sliding window of 50 SNPs (--homozyg-window-snp 50) was used to scan the genome, allowing a maximum of one heterozygous SNP per window (--homozyg-window-het 1) and five missing genotypes per window (--homozyg-window-missing 5). Genome-wide homozygous segments were identified using a minimum scanning-window threshold of 5% (--homozyg-window-threshold 0.05). Additional constraints were then applied to define final ROH regions, including a minimum ROH length of 1 Mb, a minimum of 100 SNPs per ROH (--homozyg-snp 100), a minimum SNP density of one SNP per 50kb, and a maximum gap of 1 Mb between consecutive SNPs (--homozyg-gap 1,000). These criteria were selected to minimize false-positive ROH calls while maintaining sensitivity for detecting autozygous regions. These ROH detection parameters were selected based on previous canine and livestock genomic studies and are consistent with commonly used criteria for identifying autozygous genomic regions while minimizing false-positive ROH calls (Howrigan et al., 2011; Peripolli et al., 2017). These steps collectively ensured accurate detection of ROH across the genomes of the studied dog breeds. The length of the ROH segments were grouped into five class intervals involved (I) 1–5 (Very short) (II) 5–10 (Short) (III) 10–15 (Medium) (IV) 15–20 (Long), and (V) > 20 (Very long). For each class interval, the total number and the proportion of ROH were computed, together with their mean and standard deviation (SD). The genomic coverage was also calculated using the following formula:

$$Coverage=\frac{Total\;Length\;of\;ROH\;in\;Interval}{Size\;of\;Canine\;Genome\;\left(2353.54\;MB\right)\times \#\;of\;Dogs\;\left(N=1,005\;or\;N=102\right)\;}$$

To estimate the percentage coverage, we first computed the total length of ROH segments within each chromosome for every individual dog. Next, we divided this variable by the corresponding chromosome length and finally aggregated the data by chromosome.

$$Coverage\left(F_{ROH}\right)=\frac{Total\;Length\;of\;ROH\;in\;Individual\;Dog}{Size\;of\;Canine\;Genome\;\left(2353.\;54\;MB\right)\;}$$

The fraction of the genome in runs of homozygosity for each individuls as inbreeding coefficient was calculated as:

$$F_{{ROH}_{j}}=\frac{\sum_{k}length\left({ROH}_{k}\right)}{L}$$

where ROH_k_ is the k-*th* ROH in individual j’s genome and L is the total length of the genome (or X-chromosome). As the sample population for the Labrador Retriever is ∼10x larger than the Rottweiler we adjusted the number of Labradors to be equal to the number of Rottweilers by random sampling and repeated the ROH analysis. The difference in variability of the ROH coverage was compared using a two-sample Student’s t-test.

Because the Rottweiler analysis was imbalanced with 80 cases and only 22 controls, we conducted a similar analysis on synthetically generated Rottweiler data. Using the *Imbalanced Learn* package in Python, a naïve random oversampling was performed. The newly created observations inherit the randomized characteristics of available samples. Our Rottweiler analysis was then repeated.

Upon conducting a test for equality of proportions without Yates’ continuity correction, we found that ACL rupture prevalence was significantly different between Labradors and Rottweilers (*P*-value = 2.071 x 10^−11^) (Supplementary Table S1). Specifically, Labrador Retriever dogs were between 26% and 43% less likely to experience ACL rupture than Rottweiler dogs. Consequently, we included dog breed as a covariate in our joint-dataset logistic regression model. We constructed over 30 models (10 per homozygosity parameter) based on the joint Labrador-Rottweiler dataset. The predictors varied across each model where we included: (a) only the homozygosity parameter (HP); (b) the HP and sex; (c) the HP, sex, and the interaction between sex and HP; (d) the HP and breed; (e) the HP, breed, and the interaction between breed and HP; (f) the HP, breed, and sex; (g) the HP, breed, sex, and the interaction between sex and HP; (h) the HP, breed, sex, and the interaction between breed and HP; (i) the HP, breed, sex, the interaction between sex and HP, and the interaction between breed and HP; and (j) the HP, breed, sex, and all interactions between the variables.

### Quantifying the relationship between ACL rupture risk and ROH variation

Different multiple logistic regression models were computed to assess association between homozygosity and ACL rupture in dogs. We conducted analysis using Labrador Retriever data (LAB), Rottweiler data (ROT), and a combination of Labrador and Rottweiler (LAB + ROT). We first calculated multiple measures for the analysis including total ROH counts (nROH), the sum of lengths of ROH segments (SROH), the average length of ROH segments (AVGROH) and the ROH inbreeding coefficient (F_ROH_). The AVGROH was computed by dividing the SROH with nROH for each individual dog genome. The F_ROH_ was defined as the proportion of the genome covered by ROH segments. In other words, it is the SROH over the total canine genome length, that is, 2,353.54 Mb according to the UU_Cfam_GSD_1.0 genome assembly. The model was in the form of:

$$\text{log }\left(\frac{P\text{(ACL }\text{rupture = 1)}}{\text{1 − }P\text{ (ACL rupture = 1)}}\right)=\beta_{o}+X\beta +\beta_{1}\times R$$

Where *P*(ACL rupture = 1) is the probability of ACL rupture in dogs, $\beta_{o}$ is the intercept of the model, **Xβ** represents other potential covariates included in the model such as age, sex, or interaction between terms, R is the genomic derived predictor variables as homozygosity parameters were included nROH, AVGROH, F_ROH_. Various assessment metrics were used as means to select the most appropriate model, including accuracy score, area under the curve (AUC), Akaike Information Criterion (AIC), and Bayesian Information Criterion (BIC).

## Results

### Labrador Retriever and Rottweiler genome assemblies

Raw read coverage of the genome for the Labrador Retriever and Rottweiler was 74.8 and 64.5 genome length, respectively. The breed reference genomes for the Labrador Retriever and Rottweiler had high contiguity with an N50 of >64 Mb and only 198 and 301 contigs for the Labrador Retriever and Rottweiler respectively (Table 1). Completeness of each assembly was also high with >94% of mammalian gene coverage with few duplicated, fragmented, or missing genes (Table 2).

**TABLE 1:** Reference assembly features for the Labrador Retriever and Rottweiler.

​	# Of contigs	N50 (bp)	N75 (bp)	Largest contig (bp)	Genome size (bp)
Labrador retriever					
Draft assembly	2,217	13,541,631	5,647,081	56,558,113	2,506,080,069
Final assembly	198	64,229,371	51,528,857	125,232,379	2,354,708,248
Rottweiler					
Draft assembly	2,695	11,349,818	5,332,262	51,303,492	2,467,472,345
Final assembly	301	64,091,397	51,519,555	124,942,343	2,368,062,716

bp - base pair. Genome size for canFam4.0 is 2,353.54 Mb.

**TABLE 2:** Completeness of the Labrador Retriever and Rottweiler *de novo* genome assemblies.

​	Mammalian gene coverage (%)	Complete single copy (%)	Duplicated (%)	Fragmented (%)	Missing (%)
Labrador retriever					
Draft assembly	78.7	76.5	2.2	6.6	14.7
Final assembly	94.6	92.8	1.8	1.4	4.0
Rottweiler					
Draft assembly	81.8	80.0	1.8	5.9	12.3
Final assembly	94.5	92.7	1.8	1.4	4.1

bp - base pair. Analysis was performed using BUSCOv5 (Manni et al., 2021). Genome size for canFam4.0 is 2,353.54 Mb.

### Breed-related genomic structure variation

**FIGURE 2:**
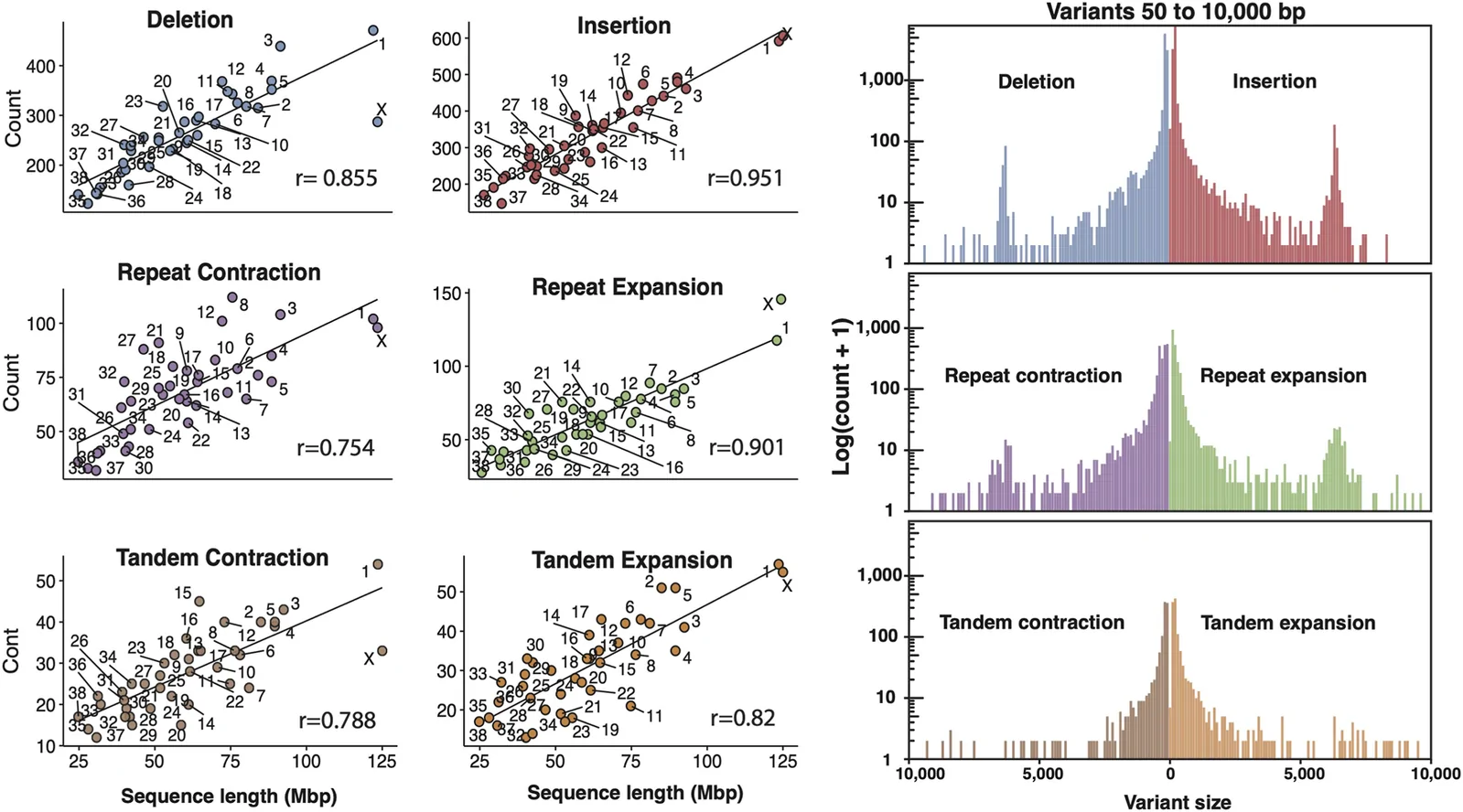
Structural variations identified using Assemblytics were categorized into six distinct classes: deletions, insertions, repeat contractions, repeat expansions, tandem contractions, and tandem expansions (left panel). The top panel shows the distribution of deletions (blue) and insertions (red) across the genome. Small to medium-sized insertions and deletions are most prevalent in the genomes with secondary peaks at ∼7kbp. The middle panel illustrates the distribution of repeat contractions (purple) and repeat expansions (green). These structural variations show a more diffuse distribution, with small variants being most common with secondary peaks again at ∼7kbp. The bottom panel presents the distribution of tandem contractions (brown) and tandem expansions (orange). Like the other variation types, these also show a clear peak of small variant size. The right panel shows a scatter plot of different structural variation counts and chromsome length for the six different variant types and the chromosomes in the genome (Chr1-Chr38, ChrX). Structural variant counts are strongly correlated with chromosome length.

In the whole genome alignment between two dog breeds, we identified 30,696 SVs across 38 autosomes and the X chromosome. SVs were categorized into six classes included deletions, insertions, repeat contractions, repeat expansions, tandem contractions, and tandem expansions with size from 50bp to 10kb. Peaks in each distribution highlight the most common sizes for each type of genetic variant, which could be critical in understanding the genomic differences contributing to phenotypic variations between the breeds. Short SVs were most frequently identified, but deletions, insertions, repeat contractions and repeat expansions also showed secondary peaks at ∼7kbp (Figure 2). We found a strong correlation between SV frequency and chromosome length for all six variant types ranged from r = 0.754 for repeat contractions to r = 0.951 for insertions.

To ensure a consistent comparison of the chromosomes, the genome was divided into fixed-size bins of 10Mbp, so that the bins (or specifically, genomic positions) were plotted on the x-axis, while the y-axis listed the 39 chromosomes (Supplementary Figure S1). We found each dog chromosome has unique SV density. While there are stable chromosomes with predominantly low-density SVs across the genome, there are also unstable chromosomes. For instance, chromosomes 10 and 11 do not have a high SV count in any genomic positions. On the other hand, chromosomes chromosome 31 and X contained SV hotspots in positions 30-40Mbp with a count of 215 SVs, and 80-90Mbp with a count of 209 SVs, respectively, highlighting areas of potential biological variation between breeds (Figures S2–S4).

## Runs of homozygosity

As summarized in Table 3, 63,524 ROH segments were detected in the Labrador Retriever group, and on the other hand, we detected 11,956 ROH segments among the Rottweiler group. Comparing the two breeds, the distribution of the length of ROH segments was similar across different length groups. Most segments in Labradors and Rottweilers were 1-5Mbp long, while the least number of ROH were within the 15–20Mb range. Genome coverage was highest in the 5–10Mb interval in the Rottweiler group. However, a higher proportion of shorter ROH segments was found in the Labrador Retriever, whereas the Rottweiler exhibited a higher percentage of longer ROH segments, indicating more recent inbreeding. Despite this, the ROH length for the Labrador Retriever group ranged up to 88.7Mbp, which is 14.3Mbp longer than the longest segment found in the Rottweiler group. In Labrador Retrievers, the total percentage of genome covered by ROH was 16.45%, mostly coming from the ROH segments of lengths between 1-5Mbp. The Rottweiler group had almost double the genome length with ROH at 32.36%.

**TABLE 3:** Runs of homozygosity analysis in the Labrador Retriever and Rottweiler.

Breed	ROH length (Mbp)	Count	Proportion (%)	Mean length (SD) (Mbp)	Coverage (%)
Labrador (n = 1,005)	1-5	38,895	61.23	2.97 (0.95)	4.88
	5-10	15,230	23.98	6.99 (1.41)	4.50
	10-15	4,913	7.73	12.07 (1.42)	2.51
	15-20	2,084	3.28	17.15 (1.41)	1.51
	*>*20	2,402	3.78	30.00 (10.76)	3.05
Rottweiler (n = 102)	1-5	6,916	57.85	2.99 (0.96)	8.61
	5-10	3,144	26.30	7.02 (1.39)	9.19
	10-15	1,019	8.52	12.19 (1.40)	5.17
	15-20	402	3.36	17.21 (1.45)	2.88
	*>*20	475	3.97	32.90 (14.15)	6.51

**FIGURE 3:**
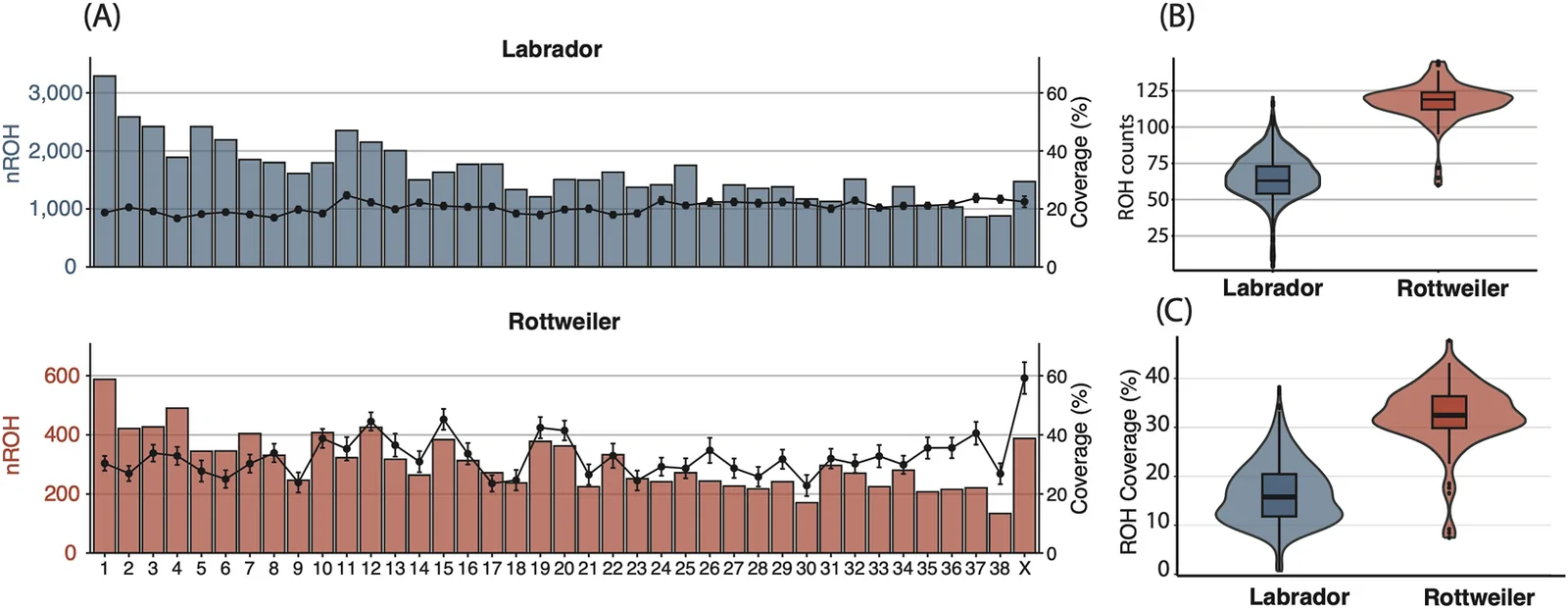
**(A)** The dual-axis chart provides a detailed comparison of the total number of ROH segments and their coverage per chromosome in the Labrador Retriever and Rottweiler populations. **(B,C)** Represent violin plot distributions for number of ROH segments and percentage of ROH coverage in Labradors and Rottweilers, with each point indicating an individual dog. Error bars show the 95% confidence interval of the percentage coverage estimate. On average, the Rottweiler genome has higher ROH coverage.

We found the Labrador Retriever has a higher number of ROH than the Rottweiler including after correction for unequal group size (Figure 3A, Supplementary Tables S2–S4). In both breeds, ROH counts generally decreased across chromosomes, with the highest number of segments being in chromosome 1 and the X chromosome, with few fluctuations in the distribution across the genome. In the Rottweiler group, we found ROH represent an increased percentage of all chromosomes compared to the Labrador Retriever. The difference between two groups was highly significant (*P* ≤ 2.2 E^-16^) using the two-sample t-test for both ROH coverage and counts (Figures 3B,C).

### Logistic regression analysis

**FIGURE 4:**
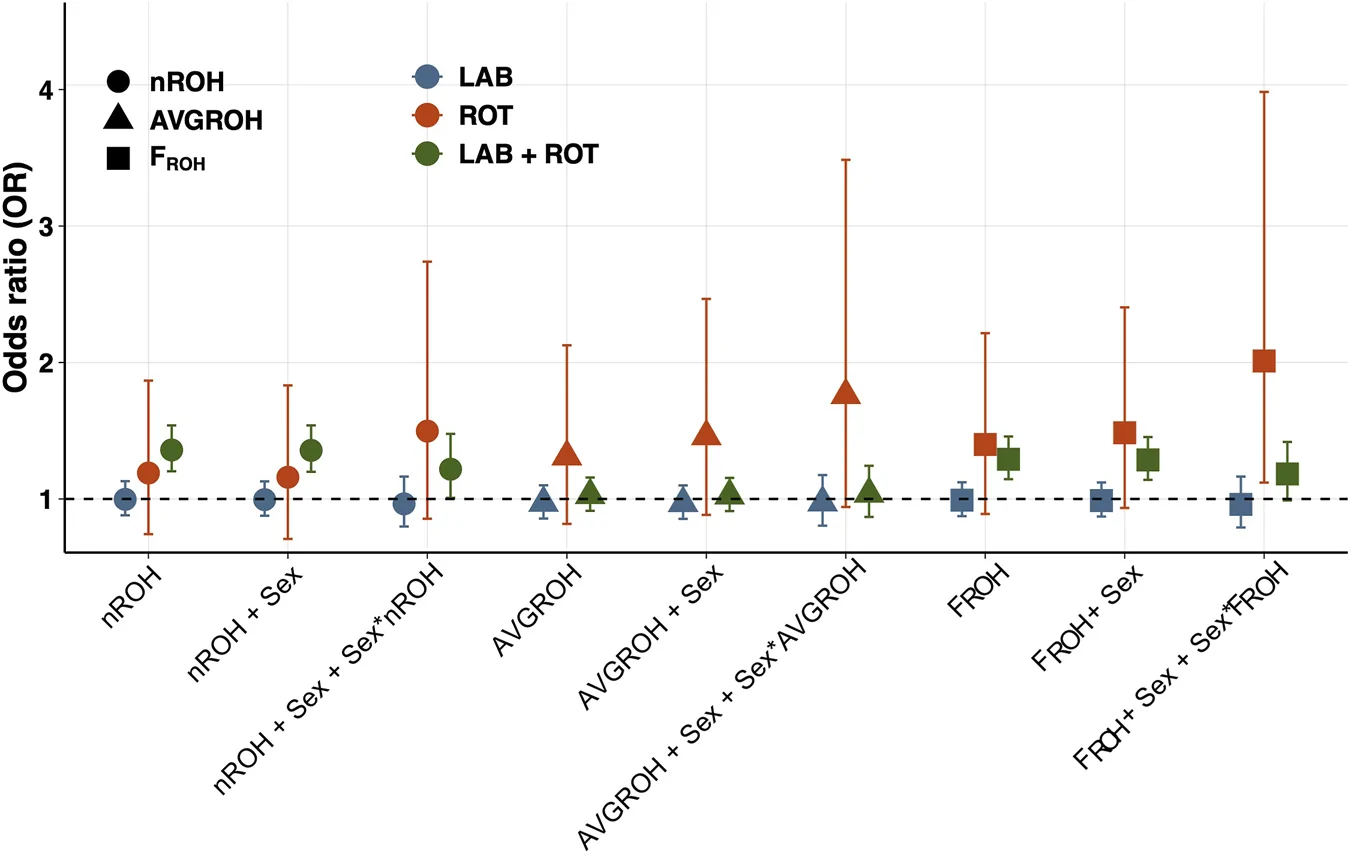
Different logistic regression models assessed the impact of three homozygosity metrics (nROH, AVGROH, and F_ROH_) on the risk of ACL rupture in the Labrador Retriever (LAB) and Rottweiler (ROT). The OR values quantify the relative increase or decrease in ACL rupture risk associated with a one-unit increase in each of the parameters. An OR greater than 1 (dashed horizontal cut-off line) indicates a positive association with higher risk, while an OR less than 1 indicates a protective effect or decreased the ACL risk. The different colors and shapes (circles, squares, and triangles) correspond to the different homozygosity parameters (nROH, AVGROH, F_ROH_) and the population used for the analysis. nROH (number of runs of homozygosity), AVGROH (average length of runs of homozygosity), and F_ROH_ (proportion of the genome covered by runs of homozygosity). Error bars represent 95% confidence intervals. The F_ROH_ + Sex + Sex*F_ROH_ model was significant (*P* = 0.026).

ACL rupture odds ratios (ORs) estimated for nROH, AVGROH, and F_ROH_ in the Labrador Retriever were all centered around 1. None of homozygosity parameters had a significant effect on ACL rupture risk in the Labrador Retriever, even when controlling for other variables and all confidence intervals include 1 (Figure 4; Supplementary Table S2). The accuracy of all nine predictive models was only marginally better than random guessing, while several models exhibit no discriminating abilities with AUC scores of below 0.5 (Supplementary Tables S2, S3).

Findings in the Rottweiler were considerably different from the Labrador Retriever. By controlling for sex and the interaction between sex and ROH coverage, we obtained a model where the coefficient of the homozygosity parameter exhibits a significant effect on ACL rupture risk (*P* = 0.026, 95% CI 1.120-3.983/unit increase in inbreeding coefficient) (Figure 4; Supplementary Table S3). We also confirmed that ACL rupture risk prediction using this model had good accuracy and AUC scores (Supplementary Tables S2, S3). After correction of imbalance between numbers of Rottweiler cases and controls, we obtained similar results and AUC score, but lowered accuracy. We also constructed over 30 models based on the joint Labrador-Rottweiler dataset (Supplementary Tables S1-S3). Supplementary Table S4 summarizes the models in which the homozygosity parameter has a significant effect on ACL rupture risk. We found inclusion of breed as a covariate was not significant in our logistic regression modeling. AVGROH as the coefficient of interest did not influence risk of ACL rupture in either breed in the all models. In the Rottweiler, the direction of the homozygosity effect on ACL rupture risk was always positive (Figure 4; Supplementary Table S3). Furthermore, in a joint analysis of the Labrador Retriever and Rottweiler groups after correction for imbalance in group size, ACL rupture risk was significantly influenced by nROH and F_ROH_ homozygosity parameters (Supplementary Table S4).

## Discussion

### Reference assemblies for the Labrador Retriever and Rottweiler breeds.

The *de novo* genome assemblies for the Labrador Retriever and Rottweiler show marked improvements in quality and completeness from the initial draft assembly to the final scaffolded version of the genome. High assembly contiguity and low gene fragmentation indicate successful assembly of high-quality genomes for these two dog breeds. The finished assemblies were like the expected genome size for canFam4.0, suggesting near-complete representation with associated high completeness with the BUSCO analysis. The work described in this study highlights the need to consider breed differences in SV in genomics research in dogs, particularly if the target breed is difference from the those of current public reference genomes.

### Genomic structural variation

We found strong positive correlations between SV frequency and chromosome length for both insertions and repeated expansions. This suggests a direct relationship where larger genomic segments are more susceptible to this type of SV. Similarly, moderate positive correlations were observed for deletions, tandem expansions, tandem contractions, and repeat contractions. These relationships generally suggested a linear relationship between SV frequency and chromosome length. Insertions and deletions were the most common types of SV identified. This may suggest underlying biological processes favor these mutations or, perhaps, technical biases in detection. Repeat expansions/contractions occurred at a lower frequency, followed by tandem expansions/contractions. This hierarchy may be due to differences in how these variations impact the genome structurally or functionally (Wang and Kirkness, 2005). Variation in correlation strengths and SV frequency suggests that different mechanisms may be driving different types of mutations, possibly due to the varying nature and impact of each type of SV on the dog genome (Edwards et al., 2021).

### Runs of homozygosity analysis

We found differences in the frequency of ROH in the two dog breeds. While both breeds exhibited some regions of reduced genetic diversity due to genetic drift or selective sweeps, the degree of inbreeding and the proportion of inbreeding caused by short versus long ROH sequences varied between two breeds, reflecting their distinct breeding histories. The higher percentage of short ROH segments in both breeds suggests recent reduction in ancient genetic diversity from inbreeding or population bottlenecks (Marsden et al., 2016; Lobo et al., 2023). The presence of longer ROH segments also suggests strong selection or historical inbreeding. In this regard, our findings suggest the Rottweiler has undergone more intense inbreeding or selection compared to the Labrador Retriever which is a more common dog breed. The Rottweiler also appears to have a more homozygous genome, likely the result of a smaller effective population size or more stringent selection pressure. Understanding the relationship between ROH and risk of ACL rupture can help inform breeding strategies aimed at reducing the prevalence of this disease by avoiding mating pairs that could produce offspring with high ROH in disease-associated regions. A higher nROH often translates into a higher percentage of coverage (McQuillan et al., 2008), although this was not the case with the Rottweiler genome in the present study.

### Logistic regression analysis

The regression analysis showed that homozygosity content has a significant impact on ACL rupture risk in the Rottweiler, but not the Labrador Retriever. This suggests the genetic contribution to ACL rupture is heterogenous in the two dog breeds, highlighting the need for breed-specific statistical models in genetic risk prediction, as genetic risk predictors may not generalize effectively across different dog breeds. The strong effects observed in Rottweilers suggest further research into breed-specific alleles or regions of the genome that are linked to cruciate ligament homeostasis is needed. However, the confidence intervals for some regression estimates in the Rottweiler analyses were relatively wide, likely reflecting the limited sample size and imbalance between cases and controls. Our analysis of the joint Labrador Retriever and Rottweiler data showed that the original multiple logistic regression modeling was reliable, although we suspect additional noise from the new samples and the randomness of the oversampling process may have affected model performance. These analyses were performed to characterize genomic differences between breeds and were not incorporated into subsequent ACL rupture risk prediction models.

Extending this analysis across the many other breeds of dog commonly affected with ACL rupture would require large-scale comparative genetic studies to identify breed-specific patterns of ROH and CNV variation (Binversie et al., 2020). GWAS focusing on ACL rupture across multiple breeds could help identify key loci where ROH or CNVs are enriched and associated with vulnerability to ligament fatigue injury. By comparing the genetic architecture of ACL rupture in breeds with a high incidence of the injury, such as Labrador Retrievers or Rottweilers, to breeds with a low incidence, we may be able to better understand how these genetic factors contribute differently to the risk of ACL rupture in various populations. In practice, for breeds with higher levels of ROH or a greater burden of harmful CNVs, breeding strategies aimed at increasing genetic diversity could help reduce the incidence of ACL rupture. Similarly, developing genetic testing to identify deleterious CNVs associated with ligament weakness could inform selective breeding programs to minimize the risk of ACL injury (Binversie et al., 2020).

## Conclusion

In summary, this study investigated the genetic factors influencing ACL rupture risk in Labrador Retrievers and Rottweilers and identified breed-specific differences in genomic homozygosity. Structural variation analyses revealed substantial genomic differences between breeds, with insertions and deletions being the most common variant types and strongly correlated with chromosome length. However, these structural variants were characterized to provide genomic context and were not directly evaluated as predictors of ACL rupture risk. ROH analyses indicated more extensive homozygosity in Rottweilers than in Labrador Retrievers. Logistic regression analyses identified a significant association between homozygosity and ACL rupture risk in Rottweilers but not in Labrador Retrievers, highlighting potential breed-specific genetic contributions to disease susceptibility. Given the relatively small and imbalanced Rottweiler dataset, these findings should be interpreted cautiously and require independent validation in larger populations. Overall, the results emphasize the importance of breed-specific approaches when investigating the genetic architecture of ACL rupture. Future studies should focus on validating these findings in independent cohorts, identifying specific genomic regions associated with disease risk, and evaluating whether ROH- or SV-based genomic features can improve genetic risk prediction for ACL rupture.

## Data Availability

The original contributions presented in the study are publicly available. The canine SNP datasets analyzed in this study can be found in the Dryad repository at https://doi.org/10.5061/dryad.47d7wm3ns.
