# Supplementary material for: Influence of homozygosity on genomic structural variation analyses for predicting ACL rupture risk in the Labrador Retriever and Rottweiler
Source: Front Genet. 2026 Sep 3;17:1875383. doi: 10.3389/fgene.2026.1875383 (PMC13581276; doi:10.3389/fgene.2026.1875383)
Supplement: Supplementary file 1 [file DataSheet1.docx]

**Table S1**. Contingency table of ACL rupture phenotypes grouped by dog breed

|  | **Case** | **Control** |
| --- | --- | --- |
| **Labrador Retriever** | 439 | 536 |
|  |  |  |
| **Rottweiler** | 80 | 22 |
|  |  |  |

**Table S2**. Logit models constructed for estimation of anterior cruciate ligament rupture in the Labrador Retriever

| Explanatory Variables | $\hat{\beta}$-coefficient (CI 95%) | *P* value | Accuracy | AUC | AIC | BIC |
| --- | --- | --- | --- | --- | --- | --- |
| nROH  nROH + Sex  nROH + Sex +  Sex x nROH  AVGROH  AVGROH + Sex  AVGROH + Sex + Sex x nROH  F_ROH_  F_ROH_ + Sex  F_ROH_ + Sex +  Sex x F_ROH_ | 0.997  (0.880 – 1.130)  0.995  (0.877 – 1.129)  0.964  (0.798 – 1.164)  0.971  (0.856 – 1.100)  0.969  (0.854 – 1.099)  0.974  (0.804 – 1.175)  0.991  (0.874 – 1.122)  0.988  (0.871 – 1.121)  0.960  (0.791 – 1.164) | 0.968  0.933  0.704  0.643  0.628  0.782  0.882  0.850  0.680 | 0.563  0.563  0.563  0.563  0.563  0.563  0.563  0.563  0.563 | 0.493  0.500  0.502  0.520  0.515  0.515  0.505  0.505  0.499 | 1381.133  1383.051  1384.861  1380.918  1382.823  1384.820  1381.112  1383.022  1384.877 | 1390.958  1397.789  1404.512  1390.743  1397.561  1404.741  1390.938  1397.760  1404.528 |

**Note**. The $\hat{\beta}$-coefficient represents the estimated odds ratio of ACL rupture risk by the respective homozygosity parameter (nROH/AVGROH/F_ROH_). CI 95% indicates 95% confidence intervals. nROH total ROH counts, AVGROH average length of ROH segments, F_ROH_ ROH inbreeding coefficient. AUC area under the receiver operator characteristic (ROC) curve, AIC Akaike Information Criterion, BIC Bayesian Information Criterion.

**Table** **S3**. Logit models constructed for estimation of anterior cruciate ligament rupture in the Rottweiler

| Explanatory Variables | $\hat{\beta}$-coefficient  (CI 95%) | *P* value | Accuracy | AUC | AIC | BIC |
| --- | --- | --- | --- | --- | --- | --- |
| nROH  nROH + Sex  nROH + Sex +  Sex x nROH  AVGROH  AVGROH + Sex  AVGROH + Sex + Sex x nROH  F_ROH_  F_ROH_ + Sex  F_ROH_ + Sex +  Sex x F_ROH_ | 1.190  (0.742 – 1.867)  1.159  (0.707 – 1.832)  1.497  (0.855 – 2.739)  1.311  (0.817 – 2.126)  1.459  (0.883 – 2.466)  1.761  (0.941 – 3.485)  1.399  (0.890 – 2.215)  1.485  (0.934 – 2.404)  2.011  (1.120 – 3.983) | 0.446  0.532  0.153  0.261  0.145  0.086  0.139  0.093  0.026 | 0.784  0.784  0.775  0.784  0.775  0.784  0.784  0.775  0.784 | 0.574  0.598  0.657  0.526  0.654  0.655  0.555  0.659  0.673 | 109.801  110.984  109.961  109.094  109.191  110.239  108.212  108.545  107.756 | 115.051  118.859  120.460  114.344  117.066  120.739  113.462  116.420  118.256 |
|  |  |  |  |  |  |  |

**Note**. The $\hat{\beta}$-coefficient represents the estimated odds ratio of ACL rupture risk by the respective homozygosity parameter (nROH/AVGROH/F_ROH_). CI 95% indicates 95% confidence intervals. nROH total ROH counts, AVGROH average length of ROH segments, F_ROH_ ROH inbreeding coefficient. AUC area under the receiver operator characteristic (ROC) curve, AIC Akaike Information Criterion, BIC Bayesian Information Criterion.

**Table S4**. Logit models constructed for estimation of anterior cruciate ligament rupture in the Labrador Retriever and Rottweiler combined

| Explanatory Variables | $\hat{\beta}$-coefficient  (CI 95%) | P value | Accuracy | AUC | AIC | BIC |
| --- | --- | --- | --- | --- | --- | --- |
| nROH  nROH + Sex  nROH + Sex +  Sex x nROH  F_ROH_  F_ROH_ + Sex  F_ROH_ + Sex +  Sex x F_ROH_ | 1.359  (1.203 – 1.539)  1.356  (1.199 – 1.539)  1.218  (1.008 – 1.477)  1.291  (1.145 – 1.458)  1.286  (1.140 – 1.454)  1.183  (0.989 – 1.418) | 1.09 x 10^-6^  1.71 x 10^-6^  0.042  3.29 x 10^-5^  4.9 x 10^-5^  0.067 | 0.580  0.575  0.573  0.582  0.575  0.581 | 0.563  0.563  0.561  0.552  0.551  0.548 | 1509.467  1511.430  1511.360  1516.689  1518.509  1518.998 | 1519.486  1526.459  1531.398  1526.708  1533.537  1539.035 |
|  |  |  |  |  |  |  |

**Note**. The $\hat{\beta}$-coefficient represents the estimated odds ratio of ACL rupture risk by the respective homozygosity parameter (nROH/AVGROH/F_ROH_). CI 95% indicates 95% confidence intervals. nROH total ROH counts, AVGROH average length of ROH segments, F_ROH_ ROH inbreeding coefficient. AUC area under the receiver operator characteristic (ROC) curve, AIC Akaike Information Criterion, BIC Bayesian Information Criterion.

**Figure S1**. The heatmap summarizes the density of the extracted structural variations across the Labrador-Rottweiler chromosomes.

**Figure S2**. The line graph zooms in on the percentage coverage of ROH per chromosome shown in Figure 1. Across all chromosomes, Rottweilers exhibit higher ROH coverage than Labradors. Interestingly, the Rottweilers’ sex chromosome shows an extreme spike in ROH, reaching almost an average 60% coverage. This warrants more investigation as there might be high levels of inbreeding or strong traces of selection on this chromosome. On the other hand, Labradors have much more stable ROH coverage, especially in the sex chromosome.

**Figure S3.** The pyramid plot displays the distribution of length of ROH segments in the two dog breeds. Both density histograms agree with Table 1 such that there are higher concentrations of shorter-length ROH segments compared to the longer-length segments. This plot also shows that the shape of the distribution is skewed for both breeds.

**Figure S4.** The scatterplots describe the relationship between the number of ROH segments and their total length, with each point indicating an individual dog. Among Labrador Retrievers, a positive linear relationship can be observed, whereby a higher total ROH length means a higher nROH. However, the relationship is not as clear among Rottweilers. There seems to be a cluster in between 600-900 MB of total ROH length and 100-125 nROH rather than a trend. It could be argued that this might arise because we have a much smaller sample size of Rottweilers and that the relationship would be the same if we reduce the sample population of Labradors. However, when we reran this analysis while limiting the number of Labradors to be equal to the number of Rottweilers, we obtained consistent results each time.

**Figure S5**. Standard Deviation (SD) trend across top percentile groups. The SD decreases steadily from the top 1% (0.0174) to the top 10% (0.013), followed by an upward trend reaching the highest value at the top 25% (0.035).
